# Rural household vulnerability and COVID-19: Evidence from India

**DOI:** 10.1371/journal.pone.0301662

**Published:** 2024-04-18

**Authors:** Junyan Tian

**Affiliations:** International Food Policy Research Institute, Washington, DC, United States of America; Public Library of Science, UNITED KINGDOM

## Abstract

The coronavirus disease 2019 (COVID-19) pandemic has affected vulnerable households’ livelihoods in developing countries. Using high-frequency phone survey data from the World Bank, we assess rural Indian households’ vulnerability and poverty status during the pandemic. Results reveal that over three-fifths of Indian rural households are vulnerable to poverty in the context of COVID-19, despite India’s evident progress in mitigating poverty in the pre-pandemic era. Poverty plays a major role in accounting for variations in household vulnerability; however, the impact of risks on household welfare is not negligible. On average, households with more members, older household heads, and more outmigrants are more vulnerable to poverty during the pandemic. The impacts of the gender of the household head, access to masks, consumption loans, and COVID-related information are nevertheless insignificant. Results stress the urgent necessity of deploying concerted interventions to strengthen household vulnerability in rural India.

## 1. Introduction

The unexpected coronavirus disease 2019 (COVID-19) pandemic has adversely affected household livelihoods in developing countries [1, 2]. Apart from its intermediate impact on public health, the pandemic has had pronounced effects on economic development and poverty mitigation [3, 4]. Governments, typically, implemented regional or national lockdowns and travel restrictions to curb the health toll resulting from COVID-19, which further hampered economic activities. Based on a recent estimate by the World Bank, roughly 100 million people have entered into poverty in 2020 owing to the outbreak of the pandemic [5]. The growing incidence of poverty is, unfortunately, unlikely to be reversed in the short run, as there is limited evidence showing a robust recovery of the labor market, supply chains, and macroeconomics in developing countries. It is, therefore, imperative to design and deploy effective interventions to strengthen household welfare vulnerability until the adverse effects of the pandemic vanish.

However, understanding the dynamics of household vulnerability is a prerequisite for designing useful instruments, especially in the context of COVID-19. While a substantial number of studies have scrutinized poverty and vulnerability dynamics in developing countries (e.g., Celidoni [6]; De la Fuente [7]; Gaiha & Imai [8]; Imai et al. [9]; Klasen et al. [10]; Kurosaki [11]; Ligon [12]; Magrini et al. [13]; Mina & Imai [14]; Parker & Kozel [15]; Swain & Floro [16]), their observations have generally been derived from a research of a prior period during which people hardly encountered shocks comparable to the pandemic. Arguably, however, poverty and vulnerability studies may need to concentrate more on the precipitous risks associated with the health shock and economic downturns during COVID-19 [1]. Hence, an immense need exists to characterize non-poor households who could easily become poor, in addition to identifying the permanent poor. A few studies have documented the likely impacts of COVID-19 on poverty [4, 17–21]. To the best of our knowledge, however, none of these studies have rigorously quantified household vulnerability in the presence of the COVID-19 shock.

Consequently, despite that the pandemic has been prevalent for three years, still, little is known about the changes in household vulnerability in light of the epidemic. Therefore, in attempting to mitigate this knowledge gap, this study utilizes the high-frequency phone survey data from the World Bank to examine whether—and to what extent—Indian rural households are vulnerable to poverty during the pandemic. We choose India as the research area due principally to the data availability. High-frequency phone surveys rarely report numerical changes in household income or expenditure, which are typically essential elements for rigorous vulnerability and poverty assessments. However, the phone survey in rural India was an exception, enabling us to quantitatively investigate the household vulnerability status during the pandemic.

Our study contributes to the emerging body of literature on COVID in several aspects. First, this study is among the few rigorous assessments of vulnerability dynamics in the context of COVID-19, which complements the previous literature on household vulnerability using pre-pandemic survey data. The empirical analysis documents that rural households in India have become highly vulnerable during the pandemic due to pronounced poverty and risks. Despite significant progress in poverty mitigation in India [22], the estimated prevalence of vulnerable households via phone survey data differs significantly from the incidence of poverty, revealing that non-poor households with an average expenditure slightly higher than the poverty line are highly likely to enter poverty during the pandemic. Second, we identify the types of rural households that are, on average, more vulnerable during the pandemic. Overall, rural households with more members, older household heads, and migrants are more vulnerable to poverty during the COVID-19 period in India. However, results also document that access to loans endorsing daily consumption and timely dissemination of COVID-19-related information does not strengthen households against vulnerability in the short term. We, thus, suggest that the Indian government should target vulnerable households and launch effective initiatives accordingly to curb the adverse effects of COVID-19 on rural poverty.

The remainder of this paper is organized as follows: Section 2 describes the research background and reviews the relevant literature. Section 3 introduces the conceptual framework and research data used in this study. Section 4 presents the vulnerability and poverty estimates and their correlates. Section 5 concludes the paper.

## 2. Context and literature review

### 2.1 COVID-19 in India

India is among the most populous countries in the world. Remarkable progress has been made in terms of economic development and poverty abatement over the last three decades. According to the World Bank, while India’s per capita income was approximately USD 529 (constant 2015 USD) at the inception of the 1990s, the figure drastically increased by 264% right before the pandemic [23]. In the meantime, India’s poverty rate has sharply reduced from 48% in 1993 to 10% in 2019, in spite of its contemporary significant population boom [24]. However, the unexpected pandemic presented a significant challenge to the Indian economy as well as to its plan for poverty elimination. Since the first infection was reported in January 2020 [25], the number of infections and deaths surged quickly. According to the World Health Organization, more than five million of the Indian population became infected and 90,000 of them passed away by September 2020 due to COVID-19 [26]. Moreover, the pandemic disproportionately affected people in India, as 60% of infections were spread across only five states. The state of Maharashtra alone contributed more than 23.2% of the total cases [26]. In response to the outbreak of the pandemic, the Indian government implemented a strict national lockdown and cross-border travel restrictions in March 2020. The stringent lockdown in India paused several essential services and business activities. These measures aimed to curb disease transmission by restricting citizens’ mobility and clustering.

### 2.2 Literature on COVID-19 and vulnerability and poverty

Several studies have attempted to understand poverty and vulnerability dynamism during the COVID-19 pandemic. Valensisi [4] utilized data from PovcalNet to simulate COVID-19’s potential adverse effects on poverty mitigation in less developed countries. Valensisi’s [4] ex-ante analysis revealed that the pandemic may drive 68 million people into poverty in the short run and, thus, erode considerable pre-pandemic efforts for poverty abatement in less developed countries. Likewise, Mahler et al. [27] employed data from multiple sources—including high-frequency phone surveys and published national household surveys—to evaluate the variation in household welfare in light of the pandemic. Mahler et al.’s [27] simulation analysis uncovered that the global poverty population would increase by approximately 90 million in relation to their counterfactual scenario in the absence of COVID-19. The difference in the estimated growth in the poverty population between Mahler et al. [27] and Valensisi [4] is partially attributable to their differential choice of the poverty line in addition to sample countries. In addition, Mahler et al. [27] suggested an exacerbation of global income inequality. Based on their ex-ante analysis, the global Gini index is likely to increase by 0.7 points in 2020, which is attributable to the outbreak of the COVID-19 pandemic.

Exploiting the difference-in-difference approach to estimate the data from nine African and Latin American countries, Bargain and Aminjonov [17] documented that the decline in work-related mobility was considerably lower in regions with a higher prevalence of poverty during the lockdown period. To abate the spread of COVID-19 virus due to social mobility, they thus underscored the necessity of enacting social protection programs in combination with lockdown policies in poor areas. Furthermore, utilizing the regression technique to estimate high-frequency phone survey data from 31 developing countries, Bundervoet et al. [18] unraveled that over three-fifths of households in selected countries experienced noticeable income loss due to the pandemic, especially for less-educated individuals. They also found that the COVID-19-induced income loss was likely to adversely impact household food security. By exploiting the difference-in-difference approach to estimate the labor force survey data from Vietnam, Dang et al. [19] discovered that the pandemic contributed to a sharp increase in unemployment in Vietnam and a notable drop in average monthly income. The income inequality also expanded in Vietnam because over 30% of workers accepted salaries lower than the minimum wage, according to Dang et al. [19]. Their results emphasized the importance of diverting greater resources to shield vulnerable workers from the adverse COVID-led shocks.

In the context of India, Gupta et al. [20] used monthly consumer pyramid household survey data collected by the Center for Monitoring Indian Economy to estimate the variation in poverty and income inequality before and after the pandemic. They discovered that the poverty rate in India increased by approximately one-third owing to the outbreak of the pandemic in urban areas. Their results also revealed a spike in income inequality as measured by the Gini coefficient. In sharp comparison, employing data from diverse sources, Bhalla et al. [28] concluded that the extreme poverty rate in India did not increase noticeably after the outbreak of the pandemic due principally to the enactment of the food subsidy policy. Hence, Bhalla et al. [28] highlighted the critical importance of social protection programs in response to pandemic-led welfare shocks. Adopting a qualitative approach, Dutta and Fischer [29] underscored the importance of local governments in promoting social programs to facilitate disease prevention and curb household welfare loss in rural India.

## 3. Materials and methods

### 3.1 Conceptual framework

To understand the consequences of risk on poor households’ livelihoods, researchers have developed multiple ways of measuring vulnerability [30–33]. Some have defined vulnerability according to expected poverty (e.g., Chaudhuri et al. [30]), whereas others have based their vulnerability measures on expected utility (e.g., Ligon & Schechter [31]). In this study, we prefer the vulnerability estimator proposed by Ligon and Schechter [31] as opposed to other measures primarily for two reasons. First, the conceptual framework of vulnerability pioneered by Ligon and Schechter [31] is (relatively) more grounded in microeconomic theory as opposed to other poverty-based vulnerability measures [13], as it addresses the (relatively) weak theoretical background of the vulnerability estimates derived from the expected values of the Foster-Greer-Thorbecke class of poverty measures [31, 33, 34]. Second, statistically, the vulnerability estimates derived from Ligon and Schechter [31]’s approach are more valid in the presence of a measurement error, despite Chaudhuri et al.’s [30] measure of vulnerability being the best practice in a stationary environment without measurement error [35]. This difference is particularly relevant to this study’s context because the quality of the expenditure and income measures from phone surveys during the COVID-19 pandemic departs from the “best practice” approach applied to in-person surveys during the pre-pandemic period [36]. Admittedly, in the context of the pandemic, carrying out large-scale in-person interviews may not be feasible due to severe concerns related to health. To account for the potential measurement error that may exist in high-frequency phone surveys, we therefore exploit Ligon and Schechter’s [31] method to assess household vulnerability in rural India.

According to Ligon and Schechter [31], household *i’s* vulnerability (V_i_) is defined as follows:

$$V_{i}=U_{i}(z)‐EU_{i}(c_{i}),$$ (1)

where U_i_ denotes a strictly increasing and weakly concave function, which maps the expenditures onto the real line. Analogous to the poverty threshold widely defined in poverty literature, z represents a consumption cutoff such that households with an expenditure no less than this number would not be deemed vulnerable. C denotes the household expenditure. However, as both current poverty status and the risks that households face with can give rise to vulnerability to poverty, we then decompose the measure of vulnerability by source (poverty vs risk) as shown in Eq (2).


$$V_{i}=[U_{i}(z)‐U_{i}(Ec_{i})]+[U_{i}(Ec_{i})‐EU_{i}(c_{i})],$$ (2)

The first bracketed term shows the difference between a concave function at the selected poverty line (z) and at the household’s expected consumption, which essentially assesses the household poverty status. The concavity of U indicates that an extra unit of expected consumption expenditure has decreasing value in poverty abatement. The second term quantifies the household *i*’s vulnerability to risk. The measure of risk in Eq (2) is consistent with the ordinal measures of risk developed by Rothschild and Stiglitz [37].

Eq (3) further decomposes the risk component into covariate and idiosyncratic risks, allowing for (relatively) clear identification of the differential impact of risks.


$$V_{i}=[U_{i}(z)‐U_{i}(Ec_{i})]+[U_{i}(Ec_{i})–EU_{i}(Ec_{i}|\bar{x_{t}})]+[EU_{i}(Ec_{i}|\bar{x_{t}})]–EU_{i}(c_{i})]$$ (3)

The suffix *t* represents time, and the term Ec_i_|$\bar{x_{t}}$ denotes the expected consumption expenditure on a set of aggregate variables $\bar{x_{t}}$. Thus, the second and third bracket terms assess household *i*’s vulnerability to covariate risk (e.g., COVID-driven national and regional lockdown) and idiosyncratic risk (e.g., illness), respectively. In practice, however, the measurement of household expenditure with error, especially in phone surveys, is not uncommon [36]. We, thus, further decompose the measure of idiosyncratic risk into the risk associated with observable household characteristics **x**_it_ and risk due to changes in unobserved factors and to measurement errors in expenditures. Hence, the household *i*’s vulnerability can be expressed as:

$$V_{i}=[U_{i}(z)‐U_{i}(Ec_{it})]+[U_{i}(Ec_{it})–EU(Ec_{it}|\bar{x_{t}})]+\{EU_{i}[Ec_{it}|\bar{x_{t}})–EU_{i}[E(c_{it}|\bar{x_{t}},x_{it}]\}+\{E[c_{it}|x_{t},x_{it}]–EU_{i}(c_{it})\},$$ (4)

where the fourth bracket term denotes the unexplained risk.

Prior to estimating household vulnerability, however, it is necessary to choose the utility function (U_i_). We express the utility function as Eq (5):

$$U_{i}(c)=\frac{c^{1-\gamma }}{1-\gamma }(\gamma >0),$$ (5)

where *γ* represents a household attitude of risk aversion. In this study, we assume *γ* = 2 to keep consistent with previous microeconomic literature. In terms of model specification for estimating vulnerability, we take the vector **x**_it_ to include household income and the principal component analysis score of food insecurity. Consistent with mainstream past studies, per capita daily expenditure is employed as a measure of consumption. In addition, as expressed in Eq (6), we parameterize the expected consumption, E(c_it_|$\bar{x_{t}}$**,x**_it_], following the specificaiton of Ligon and Schechter [31]. While ∂_*i*_ and *δ_t_* controls for household and time fixed effects, the residual term **ϵ_*it*_** represents the sum of measurement error in consumption and prediction error. The technical details for vulnerability estimation are presented in Ligon and Schechter [31].


$${\hat{c}}_{it}=\partial_{i}+\delta_{t}+\theta x_{it}+\epsilon_{it}$$ (6)

### 3.2 Analyzing the correlates of household vulnerability

We employ the ordinary least squares (OLS) method to estimate Eq (7) to investigate whether and how household characteristics are correlated with vulnerability and its components in rural India during the pandemic. **Y** denotes the vulnerability estimates and its components, namely, poverty, aggregate risk, idiosyncratic risk, and unexplained risk, which are obtained by estimating Eq (4).


$$Y_{i}=\alpha_{i}+\beta Z_{i}+\epsilon_{i}$$ (7)

In addition, **Z** represents household characteristics. Following Imai et al. [9], we control for the age of the household head and household size and their quadratic terms. Moreover, Thorat [38] showed that Hindus were noticeably more vulerable to poverty in pre-pandemic India due primarily to their heavy reliance to agriculture. We thus also introduce a dummy variable indicating the religious belief of the selected household to examine whether Hindus are more vulnerable during the pandemic. Further, we include a dummy indicates whether the household owns the land of their househoses into the regression analysis as Carter and Barrett [39] found that households with more assets are less likely to experience poverty relapse. In response to the pandemic, India quickly launched a series of measures to maintain and strengthen household livelihoods. We, therefore, introduce dummy variables indicating their access to masks, COVID-related information, government transfer, and consumption loans during the pandemic, in attempting to uncover whether those measures against COVID impact household vulnerablility. Outmigrants may serve as an important source of remittance or an income source of non-farm activities. However, the outbreak of the COVID-19 and the resulting lockdown may seriously disrupt the labor market. We also use the share of outmigrants relative to the household size as an explantory variable to investigate whether household with more migrants are more vulnerable during the pandemic.

### 3.3 Data

#### 3.3.1 High frequency phone-surveys in rural India

Effective policy responses to the adverse impacts of the pandemic depend critically on the identification and assessment of the poverty and vulnerability dynamism in the context of COVID-19. These mechanisms, however, are unlikely to be holistically captured based on pre-COVID surveys. For instance, non-poor households with an average expenditure just above the poverty threshold may encounter extreme hardships due to COVID-induced economic and health shocks. In other words, they might experience a significantly higher risk of falling into poverty in light of the pandemic, as opposed to the normal period. Nonetheless, the pre-pandemic survey data can hardly contribute to identifying those vulnerable populations. It is, therefore, necessary to utilize new survey data that closely track household welfare changes during the epidemic.

Since the outbreak of COVID, numerous international organizations have closely monitored its effects on poverty by launching high-frequency phone surveys in different countries. In India, the World Bank, Development Data Lab, John Hopkins University, and ID insight jointly conducted high-frequency phone surveys (HFPS) to mitigate the evidence gap, facilitating policymakers in formulating policies to mitigate COVID-induced shocks. The survey covered six states in India, namely, Andhra Pradesh, Bihar, Jharkhand, Madhya Pradesh, Rajasthan, and Uttar Pradesh. In total, the survey team interviewed 5,005 households by phone, despite the survey attempting to reach 11,738 households via mobile phone. As the final survey sample was compiled by multiple IDinsight projects, the sampling scheme varied slightly across states. However, to generate comparable state-level estimates based on the interviewed rural households, the survey team adopted weights to the information rendered by the sampled households [40].

Household income and consumption expenditure data are integral to understanding the dynamics of poverty and vulnerability during the COVID-19 pandemic. Meanwhile, to the best of our knowledge, the majority of the recent phone surveys only provided a categorical measure of income or consumption change, which prevents researchers from performing a more thorough assessment of the change in vulnerability status. We, therefore, only utilize the second and third surveys administered in India to carry out the vulnerability estimation because the numerical figures of household expenditure and income are only reported in these two rounds, while the first round survey did not report household income in numerical terms. The second and third survey rounds were conducted in July and September 2020, respectively. Approximately 40% of interviewees responded to both rounds of surveys.

#### 3.3.2 Description of variables

Tables 1 and 2 exhibit the description and summary statistics of the variables used in the core analysis, respectively. Similar to Amare et al. [41], most observable household characteristics remained essentially unchanged across different rounds of surveys. Approximately 16% households in the entire sample are headed by females. On average, a rural household in a selected state has six members and a household head is around 42 years old. More than half of the interviewees own the land for their housing and predominantly rely on agricultural activities for their livelihoods. The descriptive statistics highlight that only a small portion of rural residents receive masks and COVID-related information during the reference period. Likewise, credit services in rural India remain highly underdeveloped, as only around 1% of residents have access to loans supporting consumption activities. Notably, the summary statistics reveal that the average per capita expenditure was roughly lower than the latest poverty threshold (2.15 $PPP) guided by the World Bank, suggesting a prevalence of poverty in light of COVID-19. In comparison, the average per capita income is slightly higher, which is largely attributable to the influence of a few outliers with remarkably higher incomes. In addition, the aggregate food insecurity calculated by the principal component analysis approach indicates that food security in rural India is indeed worse off during the pandemic.

**Table 1:** Description of variables.

Variable	Definition
Per capita income	Household income per capita (in 2017 PPP$)
Per capita expenditure	Household consumption per capita (in 2017 PPP$)
Food insecurity index	Principal component analysis index of whether the household ran out of food (1 = yes, 0 = n o), whether at least one household member experienced hunger but did not eat (1 = yes, 0 = no), and whether at least one household member went without eating for a whole day (1 = yes, 0 = no).
AgHH	Whether the household is an agricultural household (1 = yes, 0 = no)
HHgender	Sex of the household head (1 for female, 0 for male)
HHsize	Number of household members
HHsize square	Square of the household size
HHage	Age of the household head
HHage square	Square of the age of the household head
Religion	Religion (1 = Hinduism, 0 = other religions)
Ownland	Ownership of the land of housing (1 = yes, 0 = no)
Water access	Access to private water services (1 = yes, 0 = no)
Gov transfer	Received government transfer (1 = yes, 0 = no)
Mask	Received masks from the self-help group (1 = yes, 0 = no)
Covidinfo	Access to COVID-19-related information (1 = yes, 0 = no)
Migration ratio	The ratio of net migrants within the household
Consumption loan	Access to loans in support of daily consumption (1 = yes, 0 = no)

**Table 2:** Descriptive statistics.

		Round 2			Round 3	
	N	Mean	SD	N	Mean	SD
Per capita expenditure	2,166	1.711	1.658	2,246	2.025	2.470
Per capita income	2,165	3.202	26.406	2,246	3.538	25.023
Food insecure index	2,166	0.181	1.648	2,246	0.156	1.596
AgHH	2,166	0.552	0.497	2,242	0.539	0.499
HHgender	2,166	0.164	0.371	2,246	0.153	0.360
HHsize	2,166	6.656	3.028	2,246	6.346	2.762
HHage	2,030	41.688	12.868	1,308	41.761	12.854
Religion	2,166	0.940	0.238	2,233	0.909	0.288
Ownland	2,166	0.985	0.121	2,246	0.984	0.127
Water access	2,166	0.599	0.490	2,246	0.569	0.495
Gov transfer	2,058	0.603	0.489	N/A		
Mask	2,166	0.238	0.426	N/A		
Covidinfo	2,166	0.031	0.173	N/A		
Migration ratio	1,797	0.095	3.500	1,871	0.108	4.129
Consumption loan	2,166	0.001	0.037	N/A		

Note: 1) Because none of the respondents in Rajasthan, Jharkhand, and Andra Pradesh reported their household income, observations from these three states are thus removed from the analysis. Observations with a per capita expenditure higher than the 97.5% quantile or lower than the 2.5% quantile are also dropped as these outliers may bias the vulnerability estimates. 2) “N/A” indicates that no respondents replied to that question. 3) “N” and “SD” denotes the sample size and the standard deviation, respectively.

## 4. Results

### 4.1 Vulnerability estimates

Table 3 presents the vulnerability and poverty estimates. The estimated average household vulnerability is 0.62 for rural residents in India, which is significantly greater than that reported in the most recent relevant research. For instance, using national survey data, Dang and Lanjouw [42] discovered that roughly one-fifth of Indian households were vulnerable to poverty from 2004 to 2012. The noticeable departure in the magnitude of vulnerability in India reveals the sizable perverse effect of the pandemic on household welfare beyond its health tolls. It also suggests that, in spite of the remarkable poverty mitigation during the last three decades, rural households escaping from poverty remain highly vulnerable to poverty and may easily return to it due solely to an external shock, such as COVID-19. Moreover, regional disparity in vulnerability and poverty status is also observed. In both Madhya Pradesh and Uttar Pradesh, the estimated rural households’ vulnerability is greater than 0.64, primarily due to the prevalence of poverty. By contrast, the rural households in Bihar are slightly less vulnerable.

**Table 3:** Vulnerability estimates (threshold is 2.15 $PPP, in 2017 constant price).

State	Vulnerability	Poverty	Aggregate risk	Idiosyncratic risk	Unexplained risk
Uttar Pradesh	0.649	0.437	0.006	5.69E-07	0.207
Bihar	0.539	0.333	0.005	4.53E-05	0.201
Madhya Pradesh	0.678	0.471	0.006	8.48E-05	0.201
Overall	0.617	0.410	0.006	4.66E-05	0.202

Note:1 Because none of the respondents in Rajasthan, Jharkhand, and Andra Pradesh reported their household income, it is thus infeasible to estimate the household vulnerability for these three states.m 2) Household weights are controlled for when performing the vulnerability estimation.

Poverty is found to have a decisive role in accounting for household vulnerability status even during the period of COVID-19. Overall, poverty accounts for approximately 66.1% (0.41/0.62 × 100%) of household vulnerability in rural India, based on the estimated results. Moreover, aggregate risk exhibited a considerably higher (adverse) effect on household vulnerability than idiosyncratic risk, irrespective of the state. This finding outlines the importance of the association between variations in household vulnerability and the type of shocks. The estimated results also specify that a substantial share of risks is unexplained. This observation is consistent with Magrini et al. [13], justifying the adoption of Ligon and Schechter’s [31] approach to measure vulnerability due to the measurement error of the survey data. It also resonates with the Abate et al.’s [36] finding that the quality of expenditure or income reported in high-frequency phone surveys differs from in-person interviews, the best practice typically deployed by researchers during normal periods.

### 4.2 Correlates of household vulnerability

The basedline regression results are exhibited in Table 4. In line with the agriculture’s reputation of high exposure to risk, the results show that, on average, agricultural households are 13% more vulnerable to poverty than non-agricultural households, principally because of their weaker poverty status. This stresses the importance of non-farm economic activities in India, echoing Imai et al. [9], and underscores the necessity of launching effective interventions to endorse agricultural households. Moreover, the vulnerability of agricultural households worsened significantly thanks to the aggregate risks. Nonetheless, during this period, no evident difference exists in the idiosyncratic risk between agricultural and non-agricultural households in India. While Klasen et al. [10] found that female-headed households are generally more vulnerable in Thailand, the regression results here do not reveal any notable difference in vulnerability and poverty status associated with the sex of the household head. Further, female-headed households are found to face less idiosyncratic risk during the lockdown in rural India. Consistent with Ligon and Schechter [31], we find that households with more members and older household heads tend to be more vulnerable. Those households are on average poorer and more vulnerable to aggregate and idiosyncratic risks. However, an inverse U-shaped relationship was found between household size and vulnerability. This result also holds for the age of the household head and vulnerability. The estimated coefficients of the quadratic terms of age and household size suggest that the increase in vulnerability alongside an additional unit of growth in age and household size declines. Based on the estimated parameters, however, a household would be less vulnerable if the household head is older than 44 (0.026÷(2×2.95e-04)). This finding, to some extent, echoes the empirical results of Khatun and Roy [43], which revealed that aged farmers had more diversified livelihood options as opposed to their younger counterpart in rural India. According to Khatun and Roy [43], in India, aged farmers were considered having a higher propensity of finding a job in the non-farm sector, which notably benefits the livelihood of their households.

**Table 4:** Correlates of the household vulnerability and poverty estimates.

			OLS		
	(1)	(2)	(3)	(4)	(5)
Variables	Vulnerability	Poverty	Aggregate risk	Idiosyncratic risk	Unexplained risk
AgHH	0.128**	0.129**	3.53e-04**	1.88e-05	-0.001
	(0.062)	(0.057)	(0.000)	(0.000)	(0.012)
HHgender	-3.15e-04	0.008	-2.04e-04	-4.79e-05**	-0.008
	(0.134)	(0.126)	(0.000)	(0.000)	(0.017)
HHsize	0.157***	0.155***	4.51e-04***	4.54e-06	0.002
	(0.025)	(0.024)	(0.000)	(0.000)	(0.011)
HHsize square	-0.005***	-0.006***	-1.71e-05***	1.82e-08	0.001
	(0.001)	(0.001)	(0.000)	(0.000)	(0.001)
HHage	0.026**	0.027**	7.17e-05**	1.13e-05**	-0.001
	(0.012)	(0.011)	(0.000)	(0.000)	(0.004)
HHage square	-2.95e-04**	-3.08e-04***	-8.18e-07***	-1.17e-07**	1.32e-05
	(0.000)	(0.000)	(0.000)	(0.000)	(0.000)
Religion	0.182*	0.189**	0.001**	-4.26e-06	-0.008
	(0.099)	(0.088)	(0.000)	(0.000)	(0.026)
Ownland	0.110	0.045	1.61e-04	2.99e-05*	0.065
	(0.243)	(0.212)	(0.001)	(0.000)	(0.040)
Water access	-0.104*	-0.098*	-3.09e-04**	1.28e-05	-0.005
	(0.060)	(0.056)	(0.000)	(0.000)	(0.012)
Gov transfer	-0.054	-0.047	-6.55e-05	-5.79e-05	-0.007
	(0.061)	(0.057)	(0.000)	(0.000)	(0.013)
Mask	0.104	0.083	2.90e-04*	8.92e-06	0.021
	(0.065)	(0.060)	(0.000)	(0.000)	(0.013)
Covidinfo	-0.153	-0.117	-3.69e-04	2.56e-05	-0.036*
	(0.114)	(0.103)	(0.000)	(0.000)	(0.019)
Migration ratio	0.012***	0.011***	1.93e-05***	-7.06e-07**	0.001***
	(0.000)	(0.000)	(0.000)	(0.000)	(0.000)
Consumption loan	-0.398	-0.396**	-0.001	-1.07e-04	-0.001
	(0.250)	(0.198)	(0.001)	(0.000)	(0.053)
Constant	-0.900**	-1.024***	0.001	-2.59e-04**	0.124
	(0.358)	(0.321)	(0.001)	(0.000)	(0.088)
Observations	889	889	889	889	889
R^2^	0.142	0.140	0.145	0.011	0.090
RMSE	0.671	0.621	0.002	4.5e-04	0.133

Note: 1) Robust standard errors are clustered at the household level; 2) *, **, and *** indicate statistical significance at the 10%, 5%, and 1% significance levels, respectively. 3) Household weights are controlled for when performing the OLS estimations.

A substantial share of the interviewed population are followers of Hinduism. The results reveal that households belonging to Hinduism generally are more vulnerable because of their worse poverty status and higher exposure to aggregate risk. However, there was no significant difference in exposure to idiosyncratic risk associated with religious beliefs. One possible reason that may account for the relatively worse vulnerability status of households belonging to Hinduism is their heavy dependence on agriculture for livelihoods. The result of the t-test shows that the share of agricultural households belonging to Hinduism is significantly higher compared to households with other religous belief, while agricultural households are generally more vulnerable as demonstrated by the estimated coefficients in Table 4. This finding is also consistent with the observations of Thorat [38], which explored the difference in poverty incidence by religion in pre-pandemic India. Likewise, households with access to private water services are less vulnerable because they are (relatively) wealthier and less susceptible to aggregate risk. By contrast, whether a rural household received government transfers or whether residents owned the land of their housing is orthogonal to household vulnerability and poverty status. Considering the nature of the COVID shock, it is informative to understand whether the health aid provided by the self-help group would impact household vulnerability. According to the estimated coefficients reported in Table 4, however, households receiving masks and timely information about COVID do not have a distinguishable vulnerability status compared to those who did not have access to those social assistance programs. In comparison, rural households with access to consumption loans are less likely to become poor [column (2)], which is consistent with the pre-pandemic observation from Suri et al. [44]. Meanwhile, access to consumption loans does not strengthen household vulnerability status [column (1)], owing to its insignificant association with risks [columns (3)-(5)]. While the estimated parameters of access to masks, consumption loans, and COVID-related information should not be interpreted as causal, the overall insignificant results may suggest the necessity of better targeting strategies for delivering those resources in support of vulnerable households during the pandemic. Furthermore, while Mueller et al. [45] found that consumption loans strengthen household food security via facilitating consumption smoothing in the global south during the COVID period, the empirical results highlight an urgent need to stress more on insuring against risks when delivering consumption loans in light of a widespread public health shock.

In addition, households with more outmigrants—as opposed to the total household size—are more vulnerable. Unfortunately, these households are poorer and are more vulnerable to aggregate risk. While the survey data do not enable us to explore in-depth the source of migration’s association with vulnerability during the pandemic, those migrants’ income activities are possibly interrupted owing to COVID-19 and the resulting lockdown regulations.

### 4.3 Heterogeneity analysis

In the core analysis, we assume that agricultural households are homogenous for simplicity. However, this assumption may not valid if the agricultural households are highly heterogenous in rural India. Therefore, in this sub-section, we further investigate the potential heterogenous associations between distinctive agricultural households and vulnerability in the presence of the pandemic.

We only present the estimations with the inclusion of the interaction term of agricultural household dummy and the dummy indicating the ownership of their houses, because the estimated parameters of the interaction terms of agricultural household dummy with variables indicating other household characteristics are not statistically significant at the 5% or 1% level when estimating the same model specification. The regression results displayed in Table 5 suggest that agricultural households who do not own the land of their houses are those who are substantially more vulnerable during the pandemic [column (1)], because they are evidently poorer and more suspetible to aggregate shock. This finding stresses the importance of owning fixed assets to insure against vulnerability to poverty, as suggested by Carter and Barrett [36]. It also underscores that those agricultural households without ownership of their houses should be prioritized when the government and non-profit organizations design and implement social welfare programs in rural India.

**Table 5:** Heterogenous analysis.

			OLS		
	(1)	(2)	(3)	(4)	(5)
Variables	Vulnerability	Poverty	Aggregate risk	Idiosyncratic risk	Unexplained risk
AgHH	1.160***	1.093***	0.003***	2.41e-05	0.064
	(0.240)	(0.182)	(0.001)	(0.000)	(0.088)
AgHH*Ownland	-1.044***	-0.977***	-0.003***	-5.36e-06	-0.065
	(0.246)	(0.188)	(0.001)	(0.000)	(0.089)
HHgender	0.000	0.008	-2.02e-04	-4.79e-05**	-0.008
	(0.135)	(0.126)	(0.000)	(0.000)	(0.017)
HHsize	0.163***	0.161***	4.67e-04***	4.57e-06	0.002
	(0.026)	(0.024)	(0.000)	(0.000)	(0.011)
HHsize square	-0.006***	-0.006***	-1.81e-05***	1.64e-08	0.001
	(0.001)	(0.001)	(0.000)	(0.000)	(0.001)
HHage	0.027**	0.028***	7.44e-05**	1.13e-05**	-0.001
	(0.012)	(0.011)	(0.000)	(0.000)	(0.004)
HHage square	-3.08e-04**	-3.19e-04***	-8.52e-07***	-1.17e-07**	1.25e-05
	(0.000)	(0.000)	(0.000)	(0.000)	(0.000)
Religion	0.177*	0.185**	0.001**	-4.28e-06	-0.008
	(0.099)	(0.088)	(0.000)	(0.000)	(0.026)
Ownland	0.458***	0.369***	0.001***	3.17e-05	0.087***
	(0.084)	(0.085)	(0.000)	(0.000)	(0.025)
Water access	-0.094	-0.089	-2.82e-04**	1.29e-05	-0.005
	(0.060)	(0.056)	(0.000)	(0.000)	(0.012)
Gov transfer	-0.057	-0.049	-7.35e-05	-5.79e-05	-0.007
	(0.061)	(0.057)	(0.000)	(0.000)	(0.013)
Mask	0.115*	0.093	3.19e-04**	8.97e-06	0.022
	(0.064)	(0.060)	(0.000)	(0.000)	(0.013)
Covidinfo	-0.159	-0.122	-3.84e-04	2.55e-05	-0.037*
	(0.113)	(0.103)	(0.000)	(0.000)	(0.019)
Migration ratio	0.012***	0.011***	1.95e-05***	-7.06e-07**	0.001***
	(0.000)	(0.000)	(0.000)	(0.000)	(0.000)
Consumption loan	-0.403	-0.401**	-0.001	-1.07e-04	-0.001
	(0.253)	(0.201)	(0.001)	(0.000)	(0.053)
Constant	-1.278***	-1.378***	3.13e-04	-2.61e-04**	0.100
	(0.297)	(0.272)	(0.001)	(0.000)	(0.085)
Observations	889	889	889	889	889
R^2^	0.149	0.146	0.153	0.011	0.091
RMSE	0.668	0.619	0.002	4.5e-04	0.133

Note: 1) Robust standard errors are clustered at the household level; 2) *, **, and *** indicate statistical significance at the 10%, 5%, and 1% significance levels, respectively. 3) Household weights are controlled for when performing the OLS estimations.

## 5. Conclusion

The COVID-19 pandemic is an unprecedented shock to human welfare, exposing disadvantaged populations to a pronounced risk of poverty. Yet, little has been revealed regarding the poverty and vulnerability mechanisms in the context of COVID, even though three years have passed since its outbreak. This study utilizes high-frequency phone survey data to explore the rural household vulnerability in India during the pandemic. Results reveal that, indeed, household vulnerability is more exacerbated due to the outbreak of the pandemic, compared to the findings of previous studies using pre-pandemic data. While the vulnerability is still largely explained by household poverty status, the explanatory power of risk expands in the context of the pandemic. During the pandemic, the idiosyncratic risk has more severe adverse impacts on vulnerability relative to the aggregate risk. However, there still exists an evident share of risk that remains unexplained due possibly to the measurement error of the expenditure from the phone survey.

In general, rural households with older heads, more members, net outmigrants, and substantial dependence on agriculture are more vulnerable during the pandemic. The higher vulnerability of households with heavy reliance on agriculture and with more family members and outmigrants is due principally to their, on average, more severe poverty status and higher expoure to aggregate risk. By contrast, aged household heads tend to be indications of both poorer and deeper exposure to aggregate and idiosyncratic risks. Moreover, we do not find strong evidence that access to social assistance, such as the dissemination of masks and COVID-related information, strengthens household vulnerability in rural India.

This study has multiple important policy implications for practitioners aiming to mitigate the poverty deterioration in rural India induced by COVID-19. First, the estimates of household vulnerability and poverty underscore the necessity of large-scale commitment to abating the pandemic-induced poverty shocks in India. Second, our analysis provides useful information for mapping vulnerable households in rural India. Specifically, households with older-adult heads, heavier reliance on agricultural activities, and more members and net migrants require more assistance. In particular, agricultural households who do not own the land of their housing are likely to suffer most in light of the COVID-19.

This study has several limitations. First, our results cannot be interpreted as causal, as there lacks exogenous variations in household characteristics or any interventions. Thus, future research into the causal effects of developmental interventions, such as timely vaccination, on household vulnerability and poverty mitigation will provide clearer guidance for policymakers. Also, admittedly, while we assess the household vulnerability status following the conceptual framework of Ligon and Schechter [31], our utility-based vulnerability estimates may not be considered precise point estimates due to the limited information on consumption price from the HFPS survey. Future studies extending the framework of Ligon and Schechter [31] by delving deeper into the substitutional effects across commodities and the price effects will provide valuable insights. Moreover, due to the nature of the high-frequency phone survey data, our conclusion should not be overgeneralized because phone owners might systematically differ from those without mobile phones in hand. Relatedly, owing to the limitation of data, it is worth noting that our analysis may not fully capture the possible non-linear relationship between government transfer of household vulnerability because only 60.3% of the recipients reported the amount of the transfer. Furthermore, while economies of some developed countries began recovering from 2021, poorer countries are still struggling with longer-lasting detrimental consequences on the overall economy and household livelihood. Hence, studies on long-term effects of COVID on household vulnerability to poverty in developing countries may also render valuable insights.
